# Maladaptive Aggression: With a Focus on Impulsive Aggression in Children and Adolescents

**DOI:** 10.1089/cap.2019.0039

**Published:** 2019-10-07

**Authors:** Daniel F. Connor, Jeffrey H. Newcorn, Keith E. Saylor, Birgit H. Amann, Lawrence Scahill, Adelaide S. Robb, Peter S. Jensen, Benedetto Vitiello, Robert L. Findling, Jan K. Buitelaar

**Affiliations:** ^1^ Department of Psychiatry, Division of Child & Adolescent Psychiatry, University of Connecticut Medical School, Farmington, Connecticut.; ^2^ Department of Psychiatry, Icahn School of Medicine at Mount Sinai, New York, New York.; ^3^ NeuroScience, Inc., Herndon, Virginia.; ^4^ Behavioral Medical Center—Troy, Troy, Michigan.; ^5^ Department of Pediatrics, Emory University School of Medicine, Atlanta, Georgia.; ^6^ Department of Psychiatry and Behavioral Sciences, Children's National Medical Center, Washington, District of Columbia.; ^7^ Department of Psychiatry and Behavioral Sciences, George Washington University, Washington, District of Columbia.; ^8^ Department of Psychiatry, University of Arkansas for Medical Science, Little Rock, Arkansas.; ^9^ Section of Child and Adolescent Neuropsychiatry, University of Turin, Turin, Italy.; ^10^ Department of Psychiatry and Behavioral Sciences, Johns Hopkins University, Baltimore, Maryland.; ^11^ Department of Psychiatry and Behavioral Sciences, Kennedy Krieger Institute, Baltimore, Maryland.; ^12^ Department of Cognitive Neuroscience, Donders Institute for Brain, Cognition and Behaviour, Radboud University Medical Center, Nijmegen, The Netherlands.

**Keywords:** maladaptive aggression, impulsive aggression, aggression, psychiatric disorders, neurological disorders

## Abstract

***Objective:*** Aggressive behavior is among the most common reasons for referral to psychiatric clinics and confers significant burden on individuals. Aggression remains poorly defined; there is currently no consensus on the best ways to recognize, diagnose, and treat aggression in clinical settings. In this review, we synthesize the available literature on aggression in children and adolescents and propose the concept of impulsive aggression (IA) as an important construct associated with diverse and enduring psychopathology.

***Methods:*** Articles were identified and screened from online repositories, including PubMed, PsychInfo, the Cochrane Database, EMBase, and relevant book chapters, using combinations of search terms such as “aggression,” “aggressive behavio(u)r,” “maladaptive aggression,” “juvenile,” and “developmental trajectory.” These were evaluated for quality of research before being incorporated into the article. The final report references 142 sources, published from 1987 to 2019.

***Results:*** Aggression can be either adaptive or maladaptive in nature, and the latter may require psychosocial and biomedical interventions when it occurs in the context of central nervous system psychopathology. Aggression can be categorized into various subtypes, including reactive/proactive, overt/covert, relational, and IA. IA in psychiatric or neurological disorders is reviewed along with current treatments, and an algorithm for systematic evaluation of aggression in the clinical setting is proposed.

***Conclusions:*** IA is a treatable form of maladaptive aggression that is distinct from other aggression subtypes. It occurs across diverse psychiatric and neurological diagnoses and affects a substantial subpopulation. IA can serve as an important construct in clinical practice and has considerable potential to advance research.

## Introduction

Aggressive behavior is one of the most common reasons children and adolescents are referred to psychiatric clinics, and it co-occurs with several psychiatric and neurological disorders (Connor 2002; Bambauer and Connor 2005; Jensen et al. 2007). Clinical levels of aggression in children are associated with significant individual, familial, and societal economic burdens that increase with the age of the aggressive child (Raaijmakers et al. 2011). Despite the prevalence and cost of aggression—and more than 100 years of research on the subject—it remains poorly defined in the clinical setting. Currently, a number of constructs are used to describe aggressive behavior, including symptoms (e.g., irritability or hostility) (Ramirez and Andreu 2006); diagnoses (e.g., intermittent explosive disorder [IED], disruptive mood dysregulation disorder [DMDD], oppositional defiant disorder [ODD], or conduct disorder [CD]) (American Psychiatric Association 2013); and behaviors (e.g., impulsivity) (Ramirez and Andreu 2006). This lack of well-defined nosology creates diagnostic discrepancies, which, in turn, influence the clinician's ability to devise and tailor optimal treatment strategies for the individual patient. In this review, we focus on the concept of impulsive aggression (IA) in children and adolescents and present other characterizations and frameworks of aggression for context.

Since the last comprehensive child psychiatry reviews of aggression were published, there have been several new developments in the field (Connor et al. 2006; Jensen et al. 2007). First, within child psychiatry literature, discussion of aggression has largely been supplanted by research on irritability (Pagliaccio et al. 2018; Winters et al. 2018) and classified as DMDD in the *Diagnostic and Statistical Manual of Mental Disorders, Fifth Edition* (DSM-5) (American Psychiatric Association 2013). Next, IA has been identified as a treatable indication and an unmet pharmacotherapy need (Robb et al. 2019). Finally, new formulations are under development to meet this need.

Our specific aims are to (1) discuss the definitions and categories of the aggression-related constructs currently used, including the idea of adaptive and maladaptive aggression, with an emphasis on IA; (2) provide a brief discussion of the normative developmental aspects of aggression; (3) briefly discuss the developmental neurobiology of IA; (4) present pediatric psychiatric and neurological diagnoses commonly associated with IA; and (5) review psychosocial and biological interventions, both previously used and new, for IA. Because of space limitations, our review cannot provide a discussion of aggression-related concepts such as self-injurious behaviors (SIB) or suicide, nor can it provide a detailed, in-depth focus on the neurobiology of aggression. Furthermore, because of limitations in the clinical research on pediatric aggression subtypes, some of our discussion pertains to generalized aggressive behaviors.

We conclude by suggesting that the construct of IA may be an important one to advance, both for describing behavior that presents in clinical settings and for improving the focus of treatment. A better understanding of the different types of aggression may help clinicians determine whether behaviors presented by their patients reflect natural adaptive mechanisms or a neurobiologically driven pathological condition, and in turn, facilitate more targeted identification.

## Methods

Areas of interest for advancing our understanding of maladaptive aggression (and its subsets) were identified and the relevant literature was reviewed. Articles for inclusion were screened from PubMed, PsychInfo, Scopus, the Cochrane Database, EMBase, and relevant book chapters using the search terms “aggression,” “aggressive behavio(u)r,” “maladaptive aggression,” “juvenile,” and “developmental trajectory.” The search period was from 1987 to 2019, inclusive. Of the many sources that met these criteria, 142 were selected to capture the current state of the field and are included in the article and Supplementary Data.

## Aggression and Aggression-Related Emotional Constructs

A number of aggression-related constructs are included in the discussion of aggression, which potentially creates uncertainty for patients, clinicians, and researchers. Therefore, we begin by defining common aggression-related constructs, understanding that there may be some overlap across terms (Supplementary Table S1). The following terms are prominent in the literature:

(1) Irritability refers to a heightened propensity or vulnerability to feeling angry, and has been defined as an emotional state in which an individual is “easily annoyed and provoked to anger” (Safer 2009; American Psychiatric Association 2013). It is the main characteristic of the newly developed diagnosis category, DMDD, but is present in several other disorders (American Psychiatric Association 2013; Winters et al. 2018).
(2) Like irritability, the term anger refers to an emotion. However, it is distinguished from irritability, in that anger may be the emotional component of an aggressive behavior. State anger is one possible affective component of aggressive behaviors. Trait anger is associated with the frequency, duration, and intensity of angry emotions. (Miller et al. 1996; Ramirez and Andreu 2006).
(3) Agitation has been defined as a state characterized by feelings of inner tension, with irritability and anxiety, and externalized symptoms, including excessive motor activity.
(4) Hostility refers to a negative mindset of anger and aversion toward a person or thing; it is often accompanied by a desire to do harm to another. “Hostile attribution,” a related cognition, involves interpreting ambiguous environmental stimuli as threatening, increasing risk for responding with aggressive behaviors. In sum, these terms and concepts refer to mood states or emotions that may precede or co-occur with aggressive behavior. A clear understanding of these behavioral predispositions may help in the identification and treatment of aggressive behavior in clinical practice.

## Adaptive and Maladaptive Aggression

The working concept of aggression, as described by Ramirez and Andreu, is “the delivery of any form of definite and observable *harm*-giving behavior toward any target” (Ramirez and Andreu 2006). Aggression is a central facet of the behavioral repertoire across species (Connor 2002). Adaptive aggression is defined as a behavior arising from a central nervous system (CNS) that functions optimally because of evolutionary adaptation (Connor 2002). It is a normal part of development (Connor 2002), and serves many important, easily recognizable short- and long-term goals, including resource acquisition, defense of the individual or group, and establishment of dominance in social groups. Adaptive aggressive behaviors serve many prosocial ends such as competition in academic pursuits, sports, and/or business.

However, adaptive aggression may also cause much harm and distress in society. An example is neighborhood gang violence. In gangs, groups of individuals establish a leadership hierarchy, defend “turf,” sometimes with violence, and may engage in predatory theft of resources. All of these behaviors are adaptive by the above definition, yet may clearly cause harm to individuals and society. Adaptive aggressive behaviors that threaten societal norms may require intervention from psychosocial, familial, educational, juvenile or criminal justice, or political-economic institutions in certain circumstances. However, adaptive aggression does not require, and is unlikely to show, a positive response to biomedical intervention (Connor 2002).

Maladaptive aggression may be an associated behavior of an impaired CNS that is not functioning optimally, and is more likely to occur in individuals with psychiatric or neurological illnesses. Sometimes called pathological aggression, maladaptive aggression is conceptualized as the extreme of a normal distribution of aggressive behaviors in the general population (Walters and Ruscio 2013; Waltes et al. 2016).

Maladaptive aggression occurs in response to minimal or absent provocation, and tends to be abrupt, impulsive, inappropriately intense, and frequent, and is often excessive in duration (Bambauer and Connor 2005; Jensen et al. 2007). Outside observers of a child who is vulnerable to maladaptive aggression often report that the child has “lost control,” suggesting an extreme of behavioral and emotional dysregulation (Bambauer and Connor 2005). This type of behavior is considered maladaptive compared to aggressive behaviors observed in comparison groups of nonafflicted children (Connor 2002; Bambauer and Connor 2005). When maladaptive aggression is severe, and when it occurs in the context of psychopathology, management may require biomedical therapy, in addition to psychosocial interventions (Jensen et al. 2007; Saylor and Amann 2016).

In addition to optimized/nonoptimized CNS function, the environmental context in which aggression occurs is important in determining whether aggression is expressed in an adaptive or maladaptive manner. For example, consider two scenarios with the same hypothetical subject, a child with attention-deficit/hyperactivity disorder (ADHD), walking home alone from school, who is suddenly surrounded by a youth gang intent on taunting, bullying, and then physically assaulting him. In scenario (a), the child responds with sudden, frenzied, impulsive intense aggression toward his attackers, who are momentarily taken aback. Using this brief interruption to maximal advantage, the child runs away and makes his safe escape from the gang encounter. Now consider scenario (b), in which this same child arrives home, puts off completing his homework assignments, and settles in front of an electronic, first-person shooter game to calm down. Intensely absorbed in the game, he does not hear his mother tell him to turn off the game, wash his hands, and come to dinner. She raises her voice, commanding him to turn off the game. He responds with sudden, frenzied, impulsive intense verbal threats toward his mother, throwing objects, and punching the wall. Both scenarios are examples of IA, but while the first is more adaptive in the service of individual response to a threat, the same behavior in the second scenario appears more maladaptive in the context of an ordinary request.

## Subtypes of Aggression

**FIG. 1.**
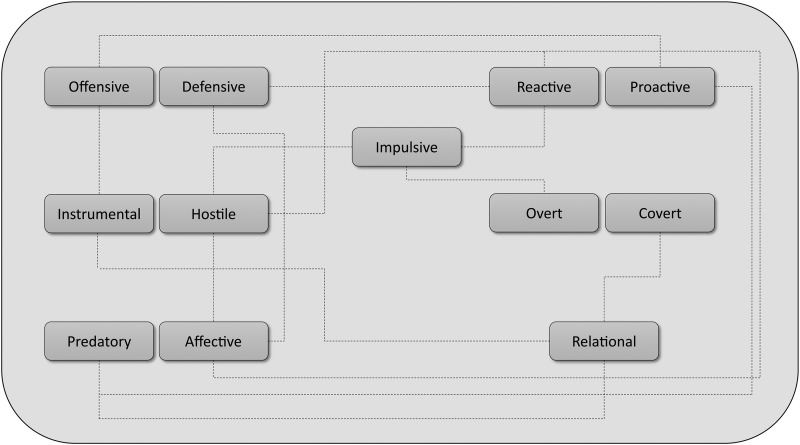
Overlapping characteristics among aggression subtypes.

Aggression can be categorized into numerous subtypes, which may be expressed in either adaptive or maladaptive ways, predominantly in a context-dependent manner, as shown above. A variety of scales and questionnaires have been developed to assess the various subtypes of aggression in clinical populations (Table 1). Aggressive behaviors are often complex and heterogeneous, and there may be varying degrees of overlap between subtypes, complicating an already intricate landscape (Fig. 1) (Connor and McLaughlin 2006). Nevertheless, the distinction between adaptive and maladaptive aggression, and among aggression subtypes that are impulsive, affective, reactive, and/or dysregulated, is heuristically useful and may foster better identification of the individuals and types of aggression that are appropriate candidates for biomedical and/or psychosocial interventions (Connor 2002). Below, we will review aggression subtypes in clinical practice and research.

**Table 1.** Some Measures of Aggression Types

*Aggression type*	*Rating scale*	*Description*	*Reporter*	*Age (years)*	*Availability*
Reactive and proactive aggression	The Reactive-Proactive Aggression Questionnaire (Raine et al. 2006)	23-item questionnaire; 12 proactive aggression items, 11 reactive aggression questions	Self	6–17	Reproduced in the open access publication (Raine et al. 2006)
Affective and predatory aggression	Vitiello Aggression Questionnaire (Vitiello et al. 1990; Vitiello and Stoff 1997)	10-item questionnaire, scoring −5 (purely affective) to +5 (purely predatory)	Caregiver	10–18	Available from the author (not in the public domain)
Hostile aggression	Aggression Questionnaire (Buss and Perry 1992; Buss and Warren 2000)	Newer version of the Buss-Durkee Hostility Inventory; 34 items assess 5 domains: physical aggression, verbal aggression, anger, hostility, and indirect aggression	Self	9–18	Available for purchase
Instrumental aggression	The Appetitive Aggression Scale (Weierstall and Elbert 2011)	15 items measuring a person's propensity toward violence-related reward	Self	13–95	Reproduced in the open access publication (Weierstall and Elbert 2011)
Overt and covert aggression	Retrospective-Modified Overt Aggression Scale (Blader et al. 2010)	16 items rated over previous week in 4 domains: verbal aggression, physical aggression toward others, aggression toward self, and destruction of property	Caregiver	6–13	Reproduced in the open access publication (Blader et al. 2010)
Relational aggression	Direct and Indirect Aggression Scale (Collett et al. 2003)	12 items assessing behaviors that covertly exploit social relationships	Self or Peer	8–15	Freely available
Impulsive aggression	Under development	15 items assessing impulsive aggression in children and adolescents with ADHD	Caregiver	6–17	Not yet available

ADHD, attention-deficit/hyperactivity disorder.

### Reactive and proactive aggression

Reactive aggression (RA) and proactive aggression (PA) are partially overlapping, yet behaviorally distinct constructs, with divergent underlying physiological hallmarks and neurological circuits (Connor 2002, 2017). RA is defined as an angry, hostile, or defensive response to frustration, provocation, or perceived threat that is rooted in the frustration-aggression model (Connor 2002; Thomson and Centifanti 2018). It is characterized by high emotional valence, autonomic nervous system arousal, and activation of fight-flight physiological mechanisms. RA can be further subcategorized according to the cause as “reactive aggression due to internal frustration” and “reactive aggression due to external provocation” (Smeets et al. 2017). In contrast, PA is a deliberate, goal-directed behavior. It is often explained by social learning theory by modeling from others and the pursuit of reward (Connor 2002). PA is characterized by overcontrolled, planned behavior accompanied by low emotional valence and low autonomic system arousal.

As discussed in detail by Bushman and Anderson (2001), some have argued that viewing RA and PA in a dichotomous manner may thwart advances in treatment. Although the dichotomy has provided the foundation for developing theories of aggression, it is now clear that aggressive acts may be more complicated than the dichotomous model implies. For example, an aggressive act may be planned and cold at the time of the occurrence, but may also be motivated by anger and the desire to harm another, as exemplified by the tragic mass murder that occurred at Columbine High School in 1999 (Bushman and Anderson 2001; Connor 2002).

### Reactive-proactive–related aggression subtypes

Subtypes of aggression related to RA and PA include predatory-affective, hostile-instrumental, and offensive-defensive aggression. The predatory and affective aggression constructs resemble PA and RA, respectively, but the predatory-affective continuum applies mainly to animal research (Connor 2002). Hostile aggression, also referred to as “affective,” “angry,” “retaliatory,” or “hot” aggression, is impulsive and angry. Instrumental aggression, also referred to as “cold” aggression, is premeditated and occurs in the absence of acute anger (Bushman and Anderson 2001; Connor 2002). Harm is not the intended goal of the behavior; rather, the aggressive act is designed to provide some reward or advantage to the aggressor (e.g., contingency reinforcement).

Hostile attribution bias is a common social-cognitive distortion that may lead to aggression. It refers to thinking that another person is responsible for some negative outcome; it has been correlated with RA, and has been described in children as young as 8 years of age (Dodge et al. 2015). This bias is predictive of acts of maladaptive RA in adulthood, suggesting that exaggeration of perceived threat may contribute to the development of chronic maladaptive aggression over time (Dodge et al. 2015). Offensive aggression is an unprovoked, instrumental behavior or attack aimed at achieving a goal, often occurring in the context of competition for social dominance or resource acquisition (Connor 2002; Veroude et al. 2016). Defensive aggression is a provoked behavior in response to an immediate threat, with the aim of reducing or eliminating the threat (Connor 2002; Veroude et al. 2016).

Although the offensive-defensive paradigm emerged from preclinical neurobiological research, it may apply to clinical research (Connor 2002; Veroude et al. 2016). For example, in a study of 369 second-grade boys and girls, evaluated again 6 years later, and then followed by examination of criminal records in young adulthood, Pulkkinen reported that general (offensive and defensive) aggression at age 8 predicted offensive aggression at age 14 and criminal convictions at age 20 (Pulkkinen 1987).

### Overt and covert aggression

Overt aggression is characterized by an open and observable response to a stimulus, such as physical fighting, property destruction, or threats of harm to others (Marsee et al. 2011; Connor 2017). This subcategory of aggression may be identified early in development. It is often initiated in the first year of life, substantially increasing in frequency with physical growth between ages 3 and 4 years, followed by a steady decline beginning at school age and continuing into adulthood (Nagin and Tremblay 2005; Olson et al. 2013). For example, about 80% of toddlers engage in some form of overt aggression (Tremblay et al. 2018). By third grade, ∼12% of children engage in hitting (the most common form of early overt aggression) (NICHD Early Child Care Research Network 2004; Olson et al. 2013).

In contrast, covert aggression is surreptitious and is exemplified by avoidance of direct confrontation, manifesting in behaviors such as stealing, cheating, vandalism, and lying; these behaviors are predictive of maladaptive aggression in adulthood (Olson et al. 2013; Connor 2017). Longitudinal data from the Oregon Youth Study, for example, showed significant intraindividual evolution in parent-rated covert antisocial behavior in boys over a 5-year period; boys who showed increases in covert antisocial behavior had relatively high levels of juvenile offenses and adult re-offense (Patterson et al. 2005; Olson et al. 2013).

### Relational aggression

Relational aggression refers to the purposeful intent to harm another through social manipulation (Björkqvist et al. 1992; Connor 2002). Also known as indirect or social aggression (Archer and Coyne 2005), this subtype of aggression is predominant in females (Björkqvist et al. 1992; Connor 2002). Examples of relational aggression include intentional peer exclusion, sharing secrets, spreading rumors or gossip, and verbal bullying (which also shares characteristics with instrumental and predatory aggression) (Björkqvist et al. 1992; Connor 2002).

Simple relational aggression is evident as early as age 3 (e.g., covering ears to ignore a peer), may become more complex in elementary/early middle school (e.g., excluding a peer), and becomes increasingly complex in adolescence (e.g., by use of social media) (Ostrov et al. 2004; Williams and Guerra 2007; Leff et al. 2010). The prevalence of relational aggression is considered moderately stable across early and middle childhood. Perpetrators tend to have additional problems, including adjustment and social processing difficulties, emotional arousal deficits, and reduction in perceived popularity.

### Impulsive aggression

IA is a maladaptive form of aggression that is reactive and overt, and occurs outside of the acceptable social context (Jensen et al. 2007; Connor 2016). In contrast to the subtypes delineated above, which can be either adaptive or maladaptive depending on the context, IA is a maladaptive expression of aggression. Characteristics include sudden, intense aggression inappropriately expressed in relationship to environmental precipitants. The individual may have frequent aggressive episodes, difficulty terminating aggression, and remorse when the episode ends. IA can be identified early in development (Lansford 2018), and the presence of this type of behavior is predictive of diverse and persistent psychopathology (Tremblay et al. 2018). It can be conceptualized as an associated feature in numerous diagnoses (Connor and McLaughlin 2006; Jensen et al. 2007; Saylor and Amann 2016).

IA has been reported to be elevated in ADHD, traumatic brain injury (TBI), autism spectrum disorder (ASD), dementia, borderline and antisocial personality disorders, psychosis, unipolar and bipolar affective disorders, substance use disorders, IED, and post-traumatic stress disorder (PTSD) (Jordan et al. 1992; Weisbrot and Ettinger 2002; Turgay 2004; Soyka 2011; American Psychiatric Association 2013; Freestone et al. 2013; Wood and Thomas 2013; Carroll et al. 2014; Ropper et al. 2014; Zhuo et al. 2014; Farmer et al. 2015; Connor et al. 2017).

Although IA is likely the most common form of aggression in clinical populations, there are currently no diagnostic criteria for IA defined in the DSM-5 (American Psychiatric Association 2013; Saylor and Amann 2016). Furthermore, there is no therapeutic agent currently indicated for the treatment of IA, although development of a therapeutic agent for the treatment of IA in children and adolescents with ADHD is ongoing. Thus, at present, there is uncertainty on the diagnostic classification and treatment of IA (Robb et al. 2019).

In sum, maladaptive aggressive behavior can manifest in a myriad of ways and be subclassified for clinical and research purposes. However, some subtypes share common attributes and regularly co-occur.

## Developmental Aspects and the Neurobiology of Aggression

Aggression is a normal part of development displayed by most children (Connor 2002). It typically occurs at a higher frequency in boys than in girls (Connor 2002). Overt aggressive behaviors (e.g., pushing, shoving, hitting, kicking, and biting) in the service of obtaining desired objects (or protecting one's desired objects from others) are common among toddlers and peak between ages 3 and 4 years (Tremblay et al. 2018). These physical behaviors begin to decrease around 5–6 years of age, as development of verbal and interpersonal skills helps to moderate aggressive impulses and facilitate more socially acceptable activities (e.g., sports competition and academic achievement). With increasing cognitive development, verbal aggression (e.g., threats and insults), relational and indirect forms of aggression (e.g., excluding a peer and malicious gossip), and covert aggressive activities (e.g., lying and cheating) increase and become more socially complex (Ostrov et al. 2004; Williams and Guerra 2007; Leff et al. 2010).

Considering reactive IA subtypes, a study of a normative sample from mid-childhood to early adolescence (starting at 7 years of age and followed annually for 6 years) identified 4 trajectory groups of RA: high stable, moderate decreasing, low increasing, and low stable (Cui et al. 2016). Over the course of development into adulthood, there is a general decrease in overt, impulsive, and reactive forms of aggression (Lansford 2018).

Discussion of the developmental neurobiology of IA is complicated by a paucity of studies on specific subtypes of aggression. Most studies focus on the development of conduct problems, antisocial behaviors, CD, ODD, callous-unemotional (CU) personality traits, and/or generalized aggressive behaviors (Klahr and Burt 2014; Noordermeer et al. 2016; Salekin 2017; Bevilacqua et al. 2018; Huesmann 2018). Furthermore, mapping the mechanisms underlying antisocial and aggressive behavior is challenging, as the behaviors arise from a complex, nonuniform, dynamic, interactive, and nonlinear interplay of heritable, biological, and cognitive factors; neuropathology; early life experience; social context; and environmental risk and protective factors across development (Meyer and Lee 2019).

The very complexity of these factors and interactions leads to etiological models of aggression that are limited in their utility for the individual practitioner and in their usefulness to clinically predict individual differences in risk for maladaptive aggression across development.

To help elucidate these complexities, we present a selective and descriptive summary of heritable, neurobiological, and environmental factors that are important in the development of aggression. Given the scarcity of studies specifically focused on IA, we cite literature from a number of conduct and antisocial behavior studies as well as general aggression literature. We specifically discuss IA where evidence is available.

### Heritable factors

A meta-analysis of twin and adoption studies reported a heritability of 65% for generalized aggressive behavior (Burt 2009). Shared environmental factors accounted for 5% and the nonshared environment accounted for 30% of the variance. Boys show higher heritability estimates than girls, especially during adolescence (Wang et al. 2013; Waltes et al. 2016). Different subtypes of aggression show different heritability estimates, with higher estimates for PA (32%–48%) than for IA (20%–43%) (Waltes et al. 2016). Developmental differences in heritability estimates are observed, as well, with the stability of preschool aggressive behaviors being mainly due to genetic factors and additional contributions from nonshared environmental factors identified as development proceeds (Lacourse et al. 2014). The strongest genetic findings on aggression stability were observed for PA (85%) compared to RA (48%) from school age to adolescence (Waltes et al. 2016).

### Neurobiology

Neurobiological factors that are important in IA include the actions of the prefrontal cortex (PFC) and its reciprocal connections with mid-brain structures involved in the acute threat response system, including the amygdala, hypothalamus, and periaqueductal gray (PAG) (Blair 2016; Bartholow 2018). In turn, these regulate the hypothalamic-pituitary-adrenal (HPA) stress response system (Walker et al. 2018). The neural circuits that appear to control aggressive responding are not specialized for this purpose alone, but support more generalized cognitive functioning such as emotional reactivity, emotional regulation, and cognitive control (Fanning et al. 2017).

Brain structures involved in the social behavior network include the anterior hypothalamic nucleus, ventromedial hypothalamus, medial amygdala, bilateral septum, PAG, and the bed nucleus of the stria terminalis (Bartholow 2018). PFC structures are thought to interact with the social-behavioral network by inhibiting or modulating their activation, allowing “top-down” control over aggressive responding (Fanning et al. 2017). A more nuanced view includes the role of the ventromedial PFC in providing information on the potential rewards and costs of future action, including aggressive responding, so that optimal response choice to environmental inputs may be achieved (Blair 2016).

In this model, IA may arise based on the recruitment of the acute threat response system, with concurrent hypofunctionality of the PFC (deficient top-down control) and enhanced cognitive expectation of reward with diminished expectations of consequence for aggressive behaviors (Rosell and Siever 2015; Blair 2016; Bartholow 2018).

The amygdala is a medial temporal lobe structure that plays an essential role in the integration of stimuli with sensory, emotional, and motivational relevance. Multiple neural connections between the amygdala and other CNS regions shape cognitive, affective, motor, and sympathetic nervous system responses to affectively and motivationally salient environmental stimuli (Rosell and Siever 2015). There exists much evidence supporting the involvement of the amygdala in fear conditioning and extinction (Marek et al. 2013), as well as in aggression (Sah 2017). For example, compared with controls, patients with maladaptive RA show increased amygdala responsiveness when exposed to threat stimuli (Blair 2010). Structural imaging studies support reduced amygdala volume, while facial expression studies indicate enhanced amygdala responsiveness in individuals with trait aggression (Rosell and Siever 2015). Imaging studies have been further consistent, with a hyporesponsive amygdala and impaired orbitofrontal cortical activity observed in psychopaths who are at risk for instrumental, proactive, and aggressive behaviors (Blair 2010). These findings suggest that overarousal of the amygdala and enhanced amygdala threat sensitivity (fear) may be associated with vulnerability to IA in a “bottom-up” model.

The striatum is composed of the caudate nucleus, putamen, and globus pallidus. The striatum integrates widespread and direct cortical inputs and modulates thalamocortical activity. As a result, the striatum plays an important role in the appropriate selection and regulation of motor, cognitive, and emotional response sequences (Rosell and Siever 2015). These structures are involved with aggressive responding through their role in goal-directed, motivational, and risk-reward information processing. These activities are modulated by the dopamine and serotonin systems, which together encode expected value and reward/risk of actions in response to environmental cues (Rosell and Siever 2015). This suggests that alterations in the functioning of the striatum may result in nonoptimized information concerning the rewards and/or consequences of IA responding.

### Neurotransmitter systems

The neurobiology of IA is complex, with many different neurotransmitters involved. One of the best-studied systems in the neurobiology of aggression is the serotonergic (5-HT) system. Strong preclinical and clinical data suggest the involvement of 5-HT receptor signaling and/or 5-HT metabolism and turnover in IA behaviors in humans (Yanowitch and Coccaro 2011; Coccaro et al. 2015; Rosell and Siever 2015).

Two hypotheses are proposed for the importance of the 5-HT system in IA. The first suggests that 5-HT stabilizes information flow in neural activity, thereby modulating reactivity to both internal and external stimuli. In this model, 5-HT serves to constrain behavior, indicating that a 5-HT deficit is associated with increased impulsivity (Spoont 1992). According to the second hypothesis, diminished net 5-HT neurotransmission leads to greater irritability, which is conceptualized as a lower threshold for responding to noxious stimuli in those with IA (Coccaro et al. 2015). Currently, 14 distinct 5-HT receptors are known and are grouped into seven main families, named 5-HT_1_ to 5-HT_7_ (Gothert 2013). 5-HT_1B_ agonists, 5-HT_2A_ antagonists, and 5-HT_2C_ agonists may help modulate IA through effects on impulsive responding (Coccaro et al. 2015).

The dopaminergic (DA) system plays a role in aggression, given its involvement in decision making, reward salience, motivation, and executive cognitive functioning (including cognitive control) (Rosell and Siever 2015). For example, the DA system is involved in the pathophysiology and psychopharmacology of ADHD, a condition often associated with IA (Gadow et al. 2014). Although research on DA and IA is limited, adequate DA availability in frontal-cortical systems may support the cognitive enhancing effects of DA, while DA systems in the striatum modulate reward processing. This suggests that greater availability of DA may protect the individual against nonadvantageous, aggressive responses to environmental frustration or provocation (Rosell and Siever 2015). Currently, five DA receptors are known: D_1_, D_2_, D_3_, D_4_, and D_5_ (Wang et al. 2018). While the D_2_ receptor is the primary target for neuroleptics and atypical antipsychotics, the D_4_ receptor may also be important in aggression (Buchmann et al. 2014).

In addition to activating the acute threat response system and fight-or-flight mechanisms that play a key role in individual survival, norepinephrine (NE) also has important functions in the PFC—especially under stressful conditions. Preclinical studies have shown that during stress, high levels of circulating catecholamines rapidly impair the top-down cognitive functions of the PFC, while strengthening the activity of the amygdala and basal ganglia (Arnsten 2009). Traumatic stress exposure may lead to dendritic atrophy in the PFC, dendritic enrichment in the amygdala, and strengthening of the NE system (Arnsten et al. 2015). High levels of NE release during conditions of traumatic stress engage alpha-1 and beta-1 adrenoceptors, which reduce the firing of PFC neurons, but strengthen neuronal activity in the amygdala (Arnsten et al. 2015). For example, in cases of child abuse, this effect on neuronal activity may result in individual hypersensitivity to cues of threat from the environment and vulnerability to dysregulated IA behaviors (Ford et al. 2011).

Other chemical and hormonal systems important in aggression include the neuropeptides arginine vasopressin (AVP) and oxytocin (OT), and the steroid hormones cortisol and testosterone. AVP has a role as a direct neuromodulator in the CNS and is thus important in the regulation of social behaviors. In preclinical research, direct administration of AVP into the hypothalamus of hamsters enhanced aggressive responding, while AVP antagonists attenuated aggression (Ferris et al. 1997). OT has an important role in the modulation of social behaviors such as affiliation, parental bonding and care of young, social communication, and anxiety-like behaviors (Kelly and Wilson 2019). Preclinical research has demonstrated an antiaggressive role for OT that appears to be complex and strongly influenced by neurobiological systems that also modulate anxiety and stress (Kelly and Wilson 2019). Cortisol and testosterone are steroid hormones that appear to influence aggression in an interdependent manner through the modulation of the amygdala's fear-or-threat neural circuits (Rosell and Siever 2015).

### Environmental factors

Heritable and neurobiological vulnerabilities to aggressive responding appear to express themselves most strongly in permissive or threatening environments (Tremblay et al. 2018). Numerous studies of children show that aggression is associated with characteristics of the social environment.

For example, one longitudinal study on the early development of chronic physical aggression found that the association between antisocial parental behaviors and those of children begins early in life, between 17 and 42 months of age (Tremblay et al. 2004). This study showed that mothers of children who became chronically aggressive were often young at the time of the child's birth, living in poverty, functioning as a single parent, had not completed high school, had smoked during pregnancy, engaged in a coercive parenting style, and/or experienced depression as a mother (Tremblay et al. 2004, 2018). Thus, the child's heritable and neurobiological vulnerabilities may interact with a dysfunctional caregiving environment early in life, influencing development of a brain that has difficulties controlling emotions and behavior (Tremblay et al. 2018).

Adverse traumatic childhood experiences, including severe stress, child abuse, and neglect, are additional potent risk factors for violent and aggressive behaviors in some individuals across the lifespan (McCrory et al. 2010; Bland et al. 2018). Studies indicate that experiencing maltreatment and adversity during early development may alter the neurobiological development and functioning of the HPA axis, hippocampus, amygdala, corpus callosum, and the PFC in ways that increase risk for psychopathology and altered threat responding, including IA (McCrory et al. 2010; Meyer and Lee 2019).

## Clinical Diagnoses Associated with IA

IA is an associated symptom of many psychiatric and neurologic disorders (Bambauer and Connor 2005; Connor and McLaughlin 2006; Connor 2017). Generally, associated IA does not denote a specific disorder, but is instead indicative of disorder severity. (Connor and McLaughlin 2006). Because disorders complicated by maladaptive aggression are numerous, in this study, we will focus on those that are most commonly observed in pediatric clinical settings.

### Attention-deficit/hyperactivity disorder

Aggression is common in children and adolescents with ADHD. In the hallmark Multimodal Treatment Study of Children with ADHD (MTA) study, for example, 54% of children with ADHD exhibited clinically significant aggression before treatment, with IA reported to be the predominant subtype (The MTA Cooperative Group 1999). In the MTA study, 26% of children whose symptoms were managed by ADHD medication exhibited persistent IA (The MTA Cooperative Group 1999; Jensen et al. 2007; Saylor and Amann 2016), demonstrating that ADHD management may not adequately address this behavior.

### Disruptive behavioral disorders

Aggression is commonly observed in children and adolescents with ODD and CD (Turgay 2004). For example, in a study of 129 children and adolescents referred for serious aggressive behavior, 93% were diagnosed with ODD and 38% with CD (Turgay 2004). CU traits, lack of remorse, and empathy deficits are all associated with increased risk for serious aggression, including the instrumental and proactive forms (Urben et al. 2018).

Blader et al. (2013) evaluated whether CU traits attenuate stimulant monotherapy in children with ADHD. Specifically, the study evaluated remission of aggression (Retrospective-Modified Overt Aggression Scale [R-MOAS] score <15) in children with ADHD and aggressive behavior as well as concomitant ODD or CD after stimulant optimization. Approximately half of the treated patients exhibited remission of aggressive behavior. However, neither CU traits nor PA was predictive of remission in children with ADHD and ODD/CD. These results suggest that even in children with PA, first-line treatment with ADHD medication is warranted, and may reduce aggression in some patients.

### Mood disorders

Although aggression was historically recognized as a way of expressing depressed mood, our understanding of mood disorders has expanded to view aggression as a co-occurring feature of the primary mood disorder. Irritability has long been seen as a symptom of depressive episodes, including the diagnosis of DMDD (Winters et al. 2018). However, aggressive behaviors—such as temper tantrums, destruction of property, and assaultive behavior—have also been observed in mixed and manic states in children with bipolar disorder (Weisbrot and Ettinger 2002; Connor et al. 2017). A study of 685 adults showed that subjects with bipolar disorder (I and II) exhibit more impulsivity and aggression/hostility over the course of their lifetimes than those with unipolar depression (Dervic et al. 2015). However, when specifically evaluating affective temperament and aggression in the euthymic state, few differences were observed (Dolenc et al. 2015), suggesting that aggression is a state-dependent, rather than a persistent, trait. Thus, differential diagnosis of irritability and aggression within mood disorder populations is essential for planning treatment and tracking response (de Aguiar Ferreira et al. 2014).

### Schizophrenia and psychosis

Psychosis is present in several disorders, including—but not limited to—schizophrenia and bipolar disorder (Khushu and Powney 2016). Although the majority of patients with psychosis are not aggressive, there is evidence of increased aggression and violence during psychotic episodes (Soyka 2011). Aggression is particularly common during first-episode psychosis, with the prevalence of violent acts estimated at 31% (16% of this patient population demonstrated “serious” aggression) (Winsper et al. 2013). Over a longer course of illness, the Danish National Birth Cohort study showed that men and women with schizophrenia demonstrated a greater likelihood of committing violent crime (odds ratios of 4.6 and 23.2, respectively) compared with normal controls (Brennan et al. 2000).

### ASD and intellectual disability

Aggression is more prevalent in patients with ASD than in the general population (Carroll et al. 2014; Farmer et al. 2015). A 2014 study categorized children with ASD into five aggressive behavioral subtypes: “hot” aggression only, “cold” aggression only, SIB only, aggression and SIB, and nonaggressive behavior (Carroll et al. 2014), suggesting that the canonical subtypes of RA and PA exist in children with ASD. Gender strongly influences aggression in children with ASD: when subjected to an aggressive attack, boys with ASD have been shown to react more aggressively than control subjects, whereas girls with ASD react less aggressively. Farmer et al. (2015) compared the frequency and types of aggressive behavior in a clinically ascertained sample of children with ASD to a sample of clinic-referred children with a range of psychiatric disorders. Neither group was selected for aggression. Based on parent reports, children with ASD demonstrated less aggressive behavior than children clinically referred for behavioral/psychological problems without ASD. The aggression in children with ASD was more likely to be reactive than proactive (Farmer et al. 2015).

Children with intellectual disability (ID) also have an increased prevalence of IA behavior. In a 15–18-month longitudinal study of 417 children with severe ID, aggression was present in 68% of the subjects, as assessed by teachers (Davies and Oliver 2016). Impulsivity was significantly associated with aggression in these children, as well.

### Post-traumatic stress disorder

Maladaptive aggression has consistently been shown to co-occur with PTSD (Jordan et al. 1992). This association appears to be largely driven by the hyperarousal cluster of symptoms evident in PTSD and/or information processing deficits (Weber 2008).

### Tourette's syndrome

Reports show that behavioral problems, including maladaptive aggression, occur in ∼23%–40% of the population with Tourette's syndrome (TS) (Budman et al. 1998; Ropper et al. 2014; Kumar et al. 2016). Based on clinical reports, aggressive behaviors in patients with TS are characterized by rage attacks, which are episodic and explosive in nature (Budman et al. 1998; Kumar et al. 2016). These outbursts may be larger in magnitude than common temper tantrums and are associated with autonomic activation (hyperarousal) and subsequent loss of control.

### Epilepsy (ictal, peri-ictal, and post-ictal periods)

Approximately 30% of patients newly diagnosed with epilepsy and ∼50% of patients with treatment-resistant epilepsy have psychiatric disorders, cognitive impairment, and social difficulties (Lin et al. 2012; Brodie et al. 2016). Although the evidence is scarce, available data suggest that aggression occurs in ∼4%–7% of patients with epilepsy (Brodie et al. 2016). Most aggressive incidents occur during the post-ictal period (Brodie et al. 2016).

### Traumatic brain injury

Aggression is frequent following TBI. One framework that may be useful for distinguishing the type of aggression following TBI in clinical practice is to categorize it as either impulsive or episodic aggression, distinguished by the time of onset and the location of the injury (Wood and Thomas 2013; Ropper et al. 2014). In patients with TBI, IA tends to occur in the acute period postinjury and may be associated with confusion and compromised problem solving. TBI often involves damage to the orbital and medial PFC, negatively affecting regulation of the amygdala. In the chronic phases of TBI, aggression is more commonly “episodic,” which, together with IA, may be grouped under the heading of IED (as per the DSM-5). Individuals with TBI exhibit seemingly sporadic mood swings, sometimes described as a “Jekyll and Hyde” phenomenon that has been associated with electric disturbances in the temporal lobe (Wood and Thomas 2013).

## Evaluation of Aggression in the Clinical Setting

**FIG. 2.**
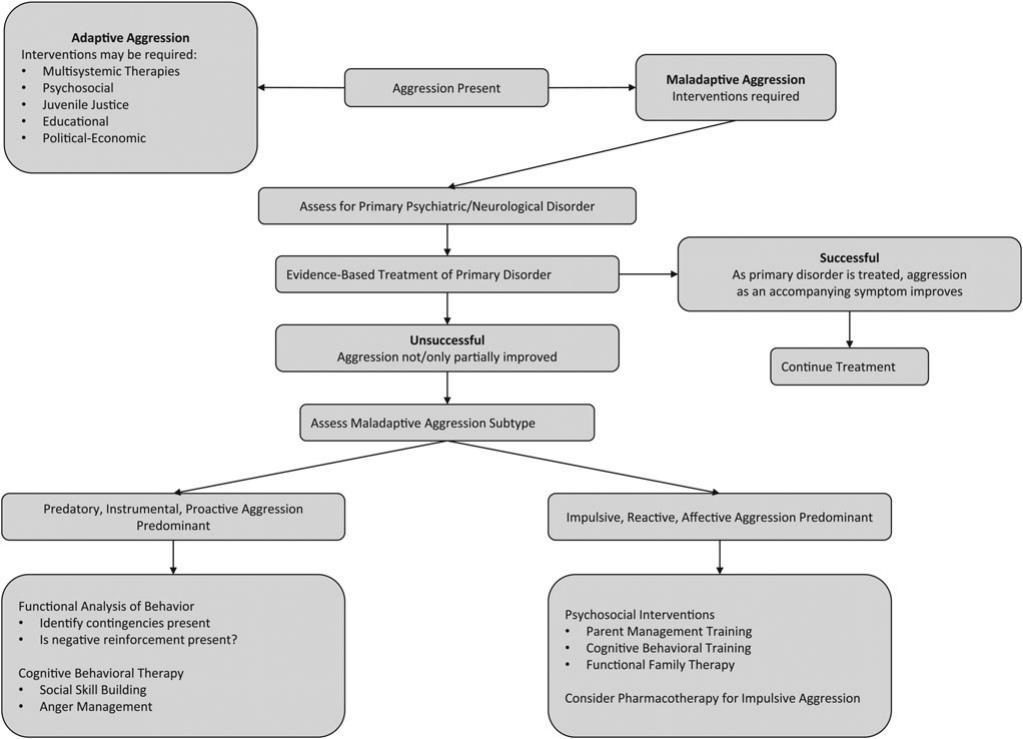
Decision-making algorithm for the assessment of aggression in clinically referred children and adolescents (Connor 2002; Connor et al. 2006; Conduct Problems Prevention Research Group 2011; Henggeler and Sheidow 2012; Dodge et al. 2015; Gurnani et al. 2016).

Guidelines for the clinical management of early-onset maladaptive aggression and IA highlight the need for thorough, systematic characterization and diagnostic evaluation of the aggressive behavior before initiating treatment (Fig. 2) (Knapp et al. 2012; Felthous and Stanford 2015). Evaluation can be considered in three steps. Step one involves recognition of maladaptive aggression. Adaptive aggression, which generally has clear and understandable objectives, does not require biomedical intervention. Maladaptive aggression requires intervention that may include psychopharmacological treatment. Assessment of contextual factors (e.g., family, school, peer group, or neighborhood) that may trigger or maintain maladaptive aggression is fundamental to treatment planning.

Following identification of maladaptive aggression, step two is to consider a psychiatric or neurological diagnosis for which maladaptive aggression is an accompanying symptom. Step three is to identify the maladaptive aggression subtype, which may guide the treatment approach. Predominantly hostile, predatory, and instrumental aggression subtypes require therapies that emphasize family intervention, behavioral treatments, cognitive behavioral therapies (CBTs), environmental monitoring, and multisystemic interventions. Aggression that is predominantly impulsive, reactive, and affective may require adjunctive pharmacotherapy, in addition to the above interventions (Connor et al. 2006).

## Treatment of Aggression

Multiple evidence-based, multisystemic treatments for aggression, antisocial behavior, and CD have been investigated and deemed effective, including Multisystemic Therapy (MST) (Henggeler and Schaeffer 2016) and the North Carolina FAST Track program (Conduct Problems Prevention Research Group 2011). These studies did not distinguish the form or function of aggressive behavior and generally report on more generalized overt aggression, conduct problems, and antisocial behaviors.

MST is a family- and community-based intervention originally developed for juvenile offenders. More recently, it has been adapted for a range of serious externalizing problems, including violent offending and juvenile substance abuse (Zajac et al. 2015). Because of the multidetermined nature of youth antisocial behaviors, MST targets concurrent risk factors at the level of the individual, family, school, and community. Outcomes on externalizing behavior are reported, but not IA specifically. Currently, 11 randomized trials and eight studies in youths with CD support the efficacy of MST (Zajac et al. 2015).

The Fast Track program interventions include parental management training, social skills training, and a universal classroom curriculum, all of which target a variety of risk factors for the development of conduct problems. Ten-year outcomes reveal reduced risk for youth antisocial outcomes compared to a nonintervention group (Pasalich et al. 2016). Specific outcomes on IA are not reported, however.

Recent reviews of psychosocial interventions and psychopharmacology for aggression are available to assist the clinician. For children and adolescents, the *Treatment of Maladaptive Aggression in Youth* (T-MAY), the *Treatment Recommendations for the Use of Antipsychotics for Aggressive Youth* (TRAAY), and the *Antisocial Behaviour and Conduct Disorders in Children and Young People: Recognition and Management National Institute for Health and Care Excellence* (NICE) guidelines provide recommendations for physicians on the treatment of maladaptive aggression (Pappadopulos et al. 2003; Knapp et al. 2012; Scotto Rosato et al. 2012; National Institute for Health and Care Excellence 2013).

IA is the most common aggression subtype seen in clinical practice (Saylor and Amann 2016). As such, the remainder of the symptom management discussion will focus on IA. Management of IA requires a personalized, multifactorial approach (Connor 2002). First-line therapy should include psychosocial interventions, adding pharmacological interventions if the former fail to curtail symptoms. Because IA often arises in the context of other diagnoses, the T-MAY and TRAAY guidelines recommend that treatment focuses on the primary disorder rather than IA (Pappadopulos et al. 2003; Scotto Rosato et al. 2012).

When aggression is particularly severe, however, it may be necessary to initiate treatment with antiaggression agents concurrent with the treatment for the primary condition (Scotto Rosato et al. 2012). Furthermore, guidelines indicate that routine use of validated scales to measure severity of aggressive symptoms is essential for accurate evaluation and treatment optimization over time (Pappadopulos et al. 2003). The T-MAY and NICE guidelines also emphasize the importance of engaging family and community in treatment programs (Scotto Rosato et al. 2012; National Institute for Health and Care Excellence 2013).

### Psychosocial interventions

Evidence-based psychosocial interventions for the treatment of maladaptive aggression, including IA, should be incorporated in a comprehensive treatment plan throughout all phases of care (Pappadopulos et al. 2003; Jensen et al. 2007; Knapp et al. 2012; Scotto Rosato et al. 2012; Gurnani et al. 2016). Psychosocial interventions include empirically supported, family-based interventions, patient-oriented techniques (such as social skills, visual and auditory interventions for those with limited language, and conflict-resolution training), parent training (e.g., reinforcing positive interactions and improving discipline strategies), teacher training (e.g., classroom management strategies), and programs targeting core deficits (Webster-Stratton et al. 2004, 2008; Kim et al. 2008; Henggeler and Sheidow 2012; Knapp et al. 2012; Maglione et al. 2012; Bearss et al. 2015).

Psychotherapy treatment approaches generally do not distinguish between types of aggression, and focus more generally on addressing physical aggression, verbal aggression, or externalizing behaviors. Core deficits targeted include anger, delay aversion, hostile attribution biases, impulsivity, emotional overarousal, and poor frustration tolerance (Sukhodolsky and Scahill 2012; Lee and DiGiuseppe 2018). To date, specific treatment approaches for IA, compared with more generalized aggression, have not been reported.

Fossum et al. (2008) conducted a meta-analysis of the literature evaluating the effects of psychosocial interventions on disruptive or aggressive behavior in children and adolescents, confirming the moderate positive effects of psychosocial interventions on maladaptive aggression. This is further supported by a more recent meta-analysis that also demonstrated moderate effects of psychological treatment in reducing parent-, teacher-, and observer-rated behavioral problems in children and adolescents with CD. A further review of meta-analyses aimed at evaluating the effects of CBT on anger control problems and aggression reported that CBT is moderately effective in reducing anger and aggression, compared to the smaller effects of other psychosocial interventions evaluated (Del Vecchio and O'Leary 2004; Saini 2009; Hofmann et al. 2012). These approaches are recommended as the primary modality of aggression management, as they have been demonstrated to be moderately effective in reducing aggressive behavior in controlled studies, with a low risk of adverse effects (Knapp et al. 2012).

### Pharmacological treatment

If psychosocial interventions are not sufficient to reduce IA, adjunctive pharmacological treatment is recommended (Fig. 2). As summarized above, the currently recommended strategy is to treat the primary disorder first (using monotherapy when possible), in conjunction with continuing psychosocial interventions (Khan et al. 2019). Psychopharmacological research specifically focused on aggression subtypes such as IA remains scarce.

Many more studies are available on the psychopharmacological treatment of aggression-related diagnoses such as CD (Hambly et al. 2016), ODD (Pringsheim et al. 2015), and ADHD-related disruptive behavior disorders (Newcorn et al. 2005). For example, the Treatment Of Severe Childhood Aggression (TOSCA) study found a moderate effect size (ES) for risperidone versus placebo when added to optimized stimulant and ongoing parent management training in children with ADHD and CD, and/or ODD (Gadow et al. 2014). Other studies have examined the effects of psychopharmacology on generalized childhood overt aggression (Pappadopulos et al. 2006). Despite a growing pediatric psychopharmacological research base on aggression-related diagnoses and constructs, however, few studies have specifically investigated aggression subtypes such as IA.

A review of the literature from 1980 to 2005 revealed 45 randomized, controlled trials that addressed the treatment of generalized overt aggression. Overall, the ES for psychiatric medications in treating aggression was 0.56. Larger effects were noted for stimulants (ES = 0.9), atypical antipsychotics (ES = 0.9), and typical antipsychotics (ES = 0.7). Lesser effects were noted in clinical trials assessing the effectiveness of antidepressants and mood stabilizers in treating maladaptive aggression (Pappadopulos et al. 2006). For treatment of irritability in patients with ASD, the only currently FDA-approved treatments are risperidone and aripiprazole (Carroll et al. 2014).

Antipsychotic use in children and adolescents has increased, in part, due to their off-label use in the treatment of maladaptive aggression and conduct problems (Kalverdijk et al. 2017). Despite this increase, there are no FDA-approved treatments for the management of IA, and there is limited information on the management of IA in patients with psychiatric and neurological disorders. In the interim, IA is increasingly treated with off-label atypical antipsychotics (Olfson et al. 2015). This is a source of growing concern, due to the potential long-term adverse effects of antipsychotic use, including weight gain and cardiometabolic dysfunction (Olfson et al. 2015; Scahill et al. 2016). This issue underscores the need for improving treatment options for patients with IA (Gurnani et al. 2016).

SPN-810, an extended-release formulation of molindone, is currently in development as a novel treatment for IA in patients with ADHD when taken in conjunction with standard ADHD treatment. A Phase 2a proof-of-concept study with immediate-release molindone demonstrated improvements in disruptive/aggressive behaviors in children with ADHD and persistent, serious conduct problems (Stocks et al. 2012). In a Phase 2b study in children with ADHD and refractory IA, SPN-810 use resulted in significant improvement from baseline in the R-MOAS versus placebo (*p* < 0.05) (Brittain et al. 2015). In this study, SPN-810 was generally well tolerated, with the most frequent adverse events being headache, sedation, and increased appetite.

Phase 3 trials with SPN-810 are ongoing (Brittain et al. 2015). Although the specific mechanism by which SPN-810 exerts effects on IA is presently unknown, emerging data suggest it functions as a D_2_-receptor antagonist and serotonin 5-HT_2B_ antagonist. In theory, these actions may help modulate impairments in decision making associated with hypothesized reduced frontal-cortical control of top-down information processing, which in turn may help regulate a disinhibited threat response neural network (Robb et al. 2019).

## Discussion

Progress in research and treatment will benefit from the development and application of consensus-driven definitions. We propose that IA is an important clinical concept because it (1) is an identifiable construct (Bambauer and Connor 2005; Raine et al. 2006); (2) appears as a similar construct across multiple common child and adolescent psychiatric diagnoses (Jensen et al. 2007); (3) appears to be measurable in the clinical setting (Jensen et al. 2007); (4) is highly correlated with symptom severity across multiple psychiatric diagnoses (Connor and McLaughlin 2006); (5) has an identifiable neurobiology that appears distinct from other forms of serious aggression such as proactive and instrumental forms of aggression, and the CU personality traits linked to psychopathy and severe CD (Blair 2016); and (6) appears more medication responsive than predatory, instrumental forms of aggression (Blader et al. 2013; Gurnani et al. 2016).

We have defined the terms maladaptive aggression and IA as having distinct meanings. We have also identified IA as a subset of maladaptive aggression, and highlighted interventions that might be required, depending on clinical presentation. Given growing concerns about off-label prescribing of psychiatric medications—especially atypical antipsychotics—to children and adolescents with aggressive behavior, the construct of IA may serve to focus pediatric psychopharmacology on an aggression subtype that is more responsive to medication (Pappadopulos et al. 2006).

However, the presented taxonomy of aggression is not without limitations. Although aggression subtypes may appear distinct at the variable level, they frequently co-occur at the patient level. Consequently, clinicians are faced with the dilemma of evaluating and treating a complex behavior with overlapping attributes. Increased agreement on the definition of maladaptive aggression (versus adaptive aggressive behaviors) and subtypes such as IA that may respond to medications can promote a better starting place for thoughtful, safe, and effective pharmacotherapy in children and adolescents. Identifying the boundaries between subtypes of aggression may also inform future research.

Controversies in the field remain to be addressed. For example, it is presently unclear if IA would best be clinically considered a categorical DSM-5 diagnosis such as IED (Coccaro et al. 2015) or a dimensional phenomenon such as the assessment of fever or pain in the medical-surgical setting (Raine et al. 2006). Furthermore, it is also unclear if IA should be addressed as a measurable symptom complex independent of diagnosis (similar to the measurement of fever/pain), or if it should be studied principally within well-defined diagnostic groups such as ADHD, bipolar disorder, psychotic disorders, ASD, and depression (Jensen et al. 2007).

The latter approach would be congruent with current expert consensus guidelines to facilitate recognizing clear indicators of treatment efficacy during randomized controlled trials (Jensen et al. 2007). This view is also supported by the FDA, given the recent designation of fast-track status for SPN-810 in the treatment of IA (United States Securities and Exchange Commission 2015; United States Securities and Exchange Commission 2016), illustrating the importance of treating this condition and addressing this unmet pharmacotherapy need (Robb et al. 2019).

## Conclusions

Further research should focus on better methods of assessing IA in the clinical setting. The validation of self- and observer-reported rating scales for the IA construct is an important first step to help address some of the issues raised above. Better methods for identifying IA will facilitate neuroimaging and neurobiological studies of the construct. This, in turn, may lead to more scientifically informed clinical research and facilitate evidence-based psychosocial and psychopharmacological interventions for IA.

## Clinical Significance

IA is expressed in many psychiatric and neurological disorders and is a common problem seen by clinicians in everyday practice. Several types of interventions for aggression are possible, based on its clinical presentation. In this article, we have defined the terms “maladaptive aggression” and “IA” as distinct constructs that may warrant different treatment approaches. We believe that the application of these definitions in clinical practice will facilitate the proper identification and treatment of IA.

## Supplementary Material

Supplemental data
