# Supplementary material for: Maladaptive Aggression: With a Focus on Impulsive Aggression in Children and Adolescents
Source: J Child Adolesc Psychopharmacol. 2019 Oct 7;29(8):576–91. doi: 10.1089/cap.2019.0039 (PMC6786344; doi:10.1089/cap.2019.0039)
Supplement: Supplemental data [file Supp_Table1-Data.pdf]

Supplementary Data

Supplementary Table S1. Aggression-Related Constructs Commonly Used in Clinical Practice and Research

Term	*Reference*	*Description/characteristics*	*Research focus*
Irritability	Stringaris et al. (2018)	Emotion: Propensity to anger easily. Associated with aberrant reward processing and bias toward threatening stimuli.	Clinical
Anger	Veenstra et al. (2018)	Emotion: One possible affective component of aggressive behaviors (state anger). Trait anger associated with frequency, duration, and intensity of angry emotions.	Clinical
Agitation	Garriga et al. (2016)	Behavior: Heightened inner tension accompanied by irritability, anxiety, and excessive motor activity.	Clinical
Hostility	Ramirez and Andreu (2006)	Emotion: Attitude of anger and/or aversion toward another.	Clinical
Hostile attribution bias	Dodge and Coie (1987)	Cognition: Threat perception where none may exist.	Clinical
Emotional impulsivity	Faraone et al. (2019)	Neurocognition: Dysregulation of emotional inhibition in the context of minimal environmental precipitants.	Clinical
Animal models of excessive aggression	de Boer (2018)	Feral Animal Model: Identification of molecular control over CNS aggression circuits and explanation of shift from adaptive aggression to maladaptive aggression.<br>Offensive Aggression: Predation of resources.<br>Defensive Aggression: In response to threat (also known as Predatory and Affective aggression).<br>Lethal violence expressed in up to 40% of mammalian species with significant phylogenetic roots.	Preclinical

CNS, central nervous system.

Supplementary References

de Boer SF: Animal models of excessive aggression: Implications for human aggression and violence. Curr Opin Psychol 19:81–87, 2018.

Dodge KA, Coie JD: Social-information-processing factors in reactive and proactive aggression in children's peer groups. J Pers Soc Psychol 53:1146–1158, 1987.

Faraone SV, Rostain AL, Blader J, Busch B, Childress AC, Connor DF, Newcorn JH: Practitioner review: Emotional dysregulation in attention-deficit/hyperactivity disorder - implications for clinical recognition and intervention. J Child Psychol Psychiatry 60:133–150, 2019.

Garriga M, Pacchiarotti I, Kasper S, Zeller SL, Allen MH, Vazquez G, Baldacara L, San L, McAllister-Williams RH, Fountoulakis KN, Courtet P, Naber D, Chan EW, Fagiolini A, Moller HJ, Grunze H, Llorca PM, Jaffe RL, Yatham LN, Hidalgo-Mazzei D, Passamar M, Messer T, Bernardo M, Vieta E: Assessment and management of agitation in psychiatry: Expert consensus. World J Biol Psychiatry 17:86–128, 2016.

Ramirez JM, Andreu JM: Aggression, and some related psychological constructs (anger, hostility, and impulsivity); some comments from a research project. Neurosci Biobehav Rev 30:276–291, 2006.

Stringaris A, Vidal-Ribas P, Brotman MA, Leibenluft E: Practitioner review: Definition, recognition, and treatment challenges of irritability in young people. J Child Psychol Psychiatry 59:721–739, 2018.

Veenstra L, Bushman BJ, Koole SL: The facts on the furious: A brief review of the psychology of trait anger. Curr Opin Psychol 19:98–103, 2018.
